# Instability caused swimming of ferromagnetic filaments in pulsed field

**DOI:** 10.1038/s41598-021-02541-3

**Published:** 2021-12-03

**Authors:** Abdelqader Zaben, Guntars Kitenbergs, Andrejs Cēbers

**Affiliations:** MMML lab, University of Latvia, Jelgavas 3, Riga, 1004 Latvia

**Keywords:** Biological physics, Fluid dynamics

## Abstract

Magnetic filaments driven by external magnetic field are an interesting topic of research in-terms of the possible bio-medical applications. In this paper, we investigated the applicability of using ferromagnetic filaments as micro swimmers both experimentally and numerically. It was found that applying a pulse wave field profile with a duty cycle of 30$\%$ induced experimentally observable swimming, which is similar to the breast stroke of micro algae. Good agreement with numerical simulations was found. Moreover, for stable continuous swimming, an initial filament shape is required to avoid transition to the structurally preferred non-swimming S-like mode.

## Introduction

The dynamics of artificial micro-swimmers has been a research topic of growing interest over the past several years. Such swimmers have been shown to be promising candidates for bio-medical applications^1^, as in targeted drug delivery^2^ and in sensing^3^. Different designs of artificial micro-swimmers have been presented in the literature which are often inspired by biological microorganisms that uses a flagella to propel, for example, helical devices controlled by a rotating magnetic field^4^ and beating flagella driven by oscillating a transverse magnetic field^5^. Here, we investigate the dynamics of a micro swimmer made from a flexible ferromagnetic particle chain and actuated by a pulse magnetic field profile, which propels by similar mechanism as Chlamydomonos Reinhardi micro algae^6^.

Magnetic filaments may be synthesised by linking para- or ferromagnetic particles by some linker in order to obtain chains similar to the chains of magnetosomes found in the magnetotactic bacteria^7^. The feasibility of such a micro-swimmer design was first investigated numerically by Belovs and Cēbers^8^. The filament propels by breaking its time reversal symmetry as a result of the buckling instability when a static magnetic field is inverted, due to the difference between the bending and relaxation times. This is similar to the well-known Euler instability of a rod under the compression^9^. The buckling of magnetic filaments is determined by the competition between the magnetic and elastic energies. The bending modulus of the filament may be determined by the deformation of links^10^ or the magnetic interaction of particles^11–13^. The buckling of magnetic rods and shells is studied in a series of works and we can mention just some of them^14,15^. The resultant deformation can be either an ‘S’ like shape, when the filament ends bend in the opposite direction or form a ‘U’ like shape^16,17^ where the filament tangent at the center of the filament aligns with the field while its ‘arms’ move toward each other. The continuous propulsion is achieved by introducing a periodic magnetic field inversion profile, which results in the filament ‘U’ like deformations of the filament under certain initial conditions^17,18^. It is important to emphasize that anisotropy of the hydrodynamic drag is essential to have self-propulsion^19^. For an infinite rod the ratio $\zeta _{\perp }/\zeta _{\parallel }$ of drag coefficients at perpendicular $\zeta _{\perp }$ and parallel $\zeta _{\parallel }$ motion to the local orientation of the filament is approximately 2^20^. For rods of finite length this ratio is smaller, and values of 1.2–1.4 are further used in the present work. Lower ratios have been experimentally estimated using resistive force theory for swimmers at low Reynolds number. For example, a ratio of 1.4 has been found C.elegans^21^, and 1.7 for sperm cells^22^ swimming far from the surface.

Previous published experimental studies for artificial micro-swimmers actuated by a magnetic field, for example as presented in^23,24^, have been focused on using para-magnetic material to form flexible chains. Here we experimentally validate that ferromagnetic filaments propel in a piecewise constant AC field in one dimension. We also discuss the conditions required to form the ‘U’ shaped configuration and ways to optimise the swimming velocity.

**Figure 1:**
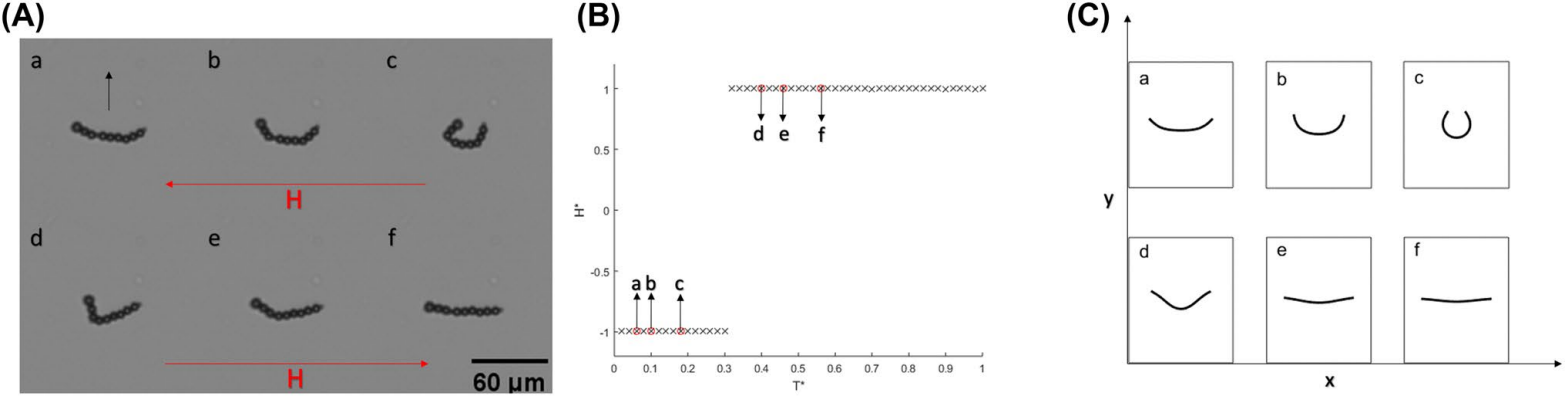
(**A**) An example of filament ‘U’ deformations under a 30$\%$ duty cycle pulse wave profile magnetic field, at different location within one period for filament with length L = 60 $\upmu$m, field frequency f = 2 Hz and field strength H = 5.2 Oe. The arrow in ‘a’ shows the swimming direction. (**B**) Magnetic field measurements vs time over one period where $H^{*} = H/H_\text {max}$ and $T^{*} = t/T$ . The red circles show the readings that correspond to images (a–f) in both (**A**) and (**C**). (**C**) Filament shapes obtained by numerical simulations which are scaled with experiments shown in (**A**), $Cm = 51$ and $T/\tau = 0.0092$.

## Results

Following the experimental procedures as presented in the “Methods” section, a static field is first applied which aligns the filament with the field, followed by a pulse wave field profile with a duty cycle of 30$\%$, where the magnetic field is inverted for 30$\%$ of the time within one period. The duty cycle 30$\%$ is found experimentally by trying different possibilities to achieve stable swimming. The choice of the definite duty cycle is based on the hypothesis of two different characteristic time scales- characteristic time of a straight filament instability growth and the characteristic time of the instability of the metastable U-like shape. A more detailed study of the last problem will be carried out in a forthcoming theoretical publication. An example of experimental filament deformation at different time *t* within one period *T* with their corresponding field reading are shown in Fig. 1A,B respectively. In Fig. 1A,a–c), the bending stage is shown. After reaching a maximum deformation in the 30$\%$ stage of the profile, the filament relaxes, as shown in Fig. 1A,d–f, until aligning with the magnetic field as shown in Fig. 1A–f. It should be noted that the lengths of the filament’s ‘arms’ are not equal, which may be the result of defects occurring during the formation process due to the non equal number of linking DNA strands along the filament. The magnetic moment is along the tangent direction as determined by the initial condition and as a result is opposite to the field when it is in the 30$\%$ part of the cycle. In this case we have the unstable regime where the filament undergoes bending due to the buckling instability. Moreover, it was found experimentally that having the unstable regime in the 70$\%$ case the filament flips its orientation where the direction of magnetization and field correspond to the stable situation.

The filament shapes obtained by numerical simulation and corresponding to the experimentally observed shapes (Fig. 1A) are shown in Fig.1C; (a–c) shows the bending stage, followed by the relaxation stage as shown in (d–f). Scaling with experimental data was done by defining two dimensionless parameters, the magnetoelastic number $Cm=MHL^{2}/A_{b}$ and T/$\tau$, where *M* is the magnetization per unit length, *H* is the magnetic field strength, *L* is the filament length, $A_{b}$ is the bending modulus, the elastic relaxation time $\tau =\zeta _{\perp } L^{4}/A_{b}$, and $\zeta _{\perp }$ is the hydrodynamic drag coefficient which is estimated by $4\pi \eta$. For the values of filament physical properties *M* and $A_{b}$ we use $M = 4.9 \pm 5.0 \times 10^{-7}$ emu and $1.5 \pm 2.0 \times 10^{-12}$  erg cm, respectively. These values were previously estimated in Ref.^25^. Moreover, we define here the anisotropy of the hydrodynamic drag coefficient $\lambda$ as − 0.2 to account for the filament flexibility and length, where $\lambda = -(\zeta _{\perp }/\zeta _{\parallel } -1)$. An example video of an experimental and numerical swimming filament can be found in the Supplementary Video S1.

This model was previously used in the numerical investigation of a ferromagnetic swimmer^8^ and a 3D study of loop formation by a ferromagnetic filament due to field inversion^17,18^. Here we modify the model to account for the piece-wise periodic field profile similar to the one defined in the experiments—a pulse wave profile with having the unstable part in the 30$\%$ of the period. Furthermore, a condition is added to the model to prevent the filament from completely relaxing and to always have a slightly curved shape before the start of the bending stage in the unstable part of the field.

**Figure 2:**
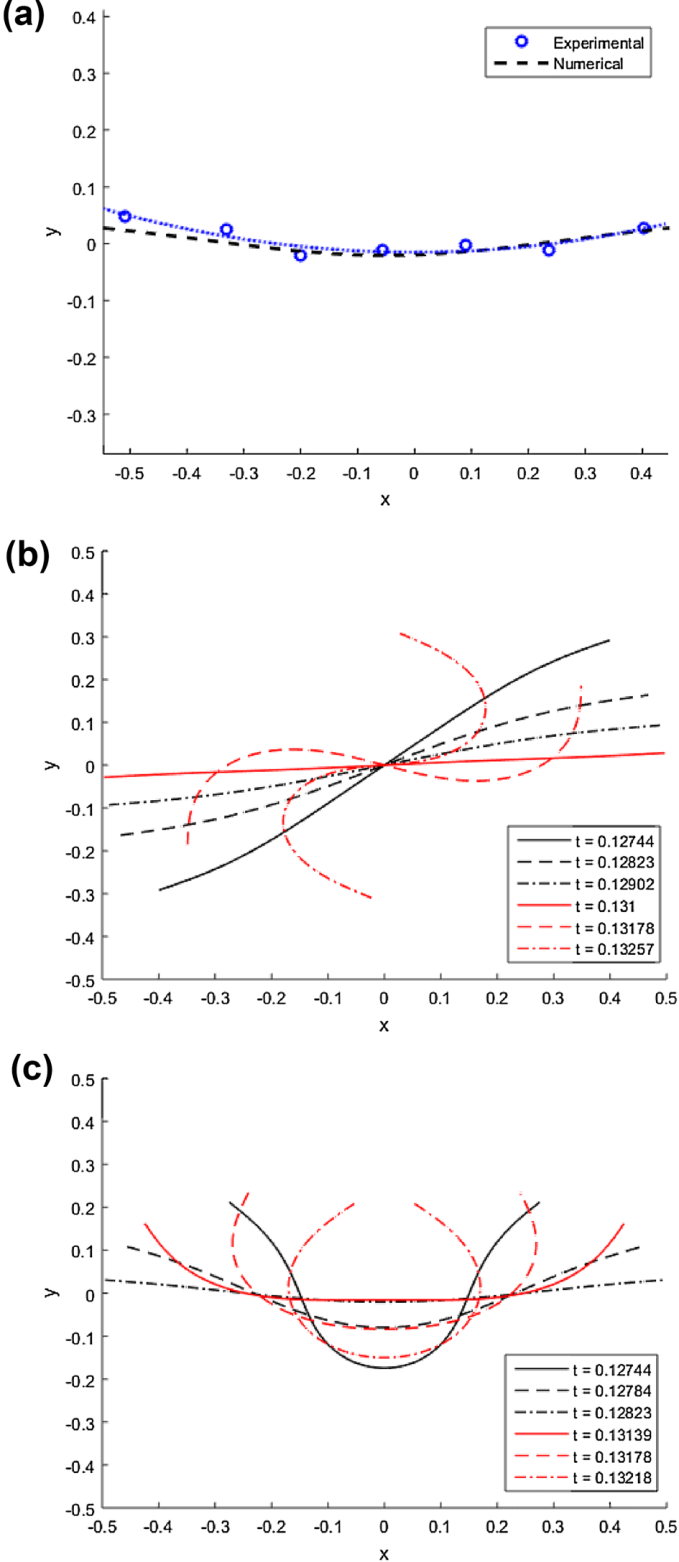
(**a**) Relaxed filament shapes obtained from experimental data and numerical simulations. For dashed line, $Cm = 30$ and $T/\tau = 0.0013$. The circles donates particle centres of experimental data with $L = 48$ $\upmu$m and $H = 5.2$ Oe. (**b**,**c**) Filament shapes obtained by numerical simulations, red and black lines donates the configuration during bending and relaxation stage respectively within one period at different time moments *t*; $Cm = 70$ and $T/\tau = 0.0052$. (**b**) and (**c**) shows the difference of results without and with the condition of curved initial shape respectively.

The initial curved shapes of the filament can be seen in Fig. 2a. This modification was made to account for the experimentally observed filament shapes where the filament was found not to completely relax and align with the magnetic field in the stable part of the field. An example of comparison between the experimental and numerical initial configurations of the filament is shown in Fig. 2a. Moreover, it was found numerically that if the condition of initial curved shape was not applied under the defined conditions similar to experiments, the ‘U’-shaped deformation could not be achieved and the filament underwent the favoured ‘S’-like deformation resulting in no propulsion. This is shown in Fig. 2b,c; in (b) the simulations were done without the initial curvature and in (c) ‘U’ was observed to have the initial filament shape before the start of the bending stage. Therefore, we propose that without an initial filament shape before the unstable part of the field, the filament will not undergo the ‘U’ shape swimming deformation mode and prefer the ‘S’ deformation mode instead. An example video of experimentally observed shapes with corresponding numerical simulations is shown in Supplementary Video S2.

**Figure 3:**
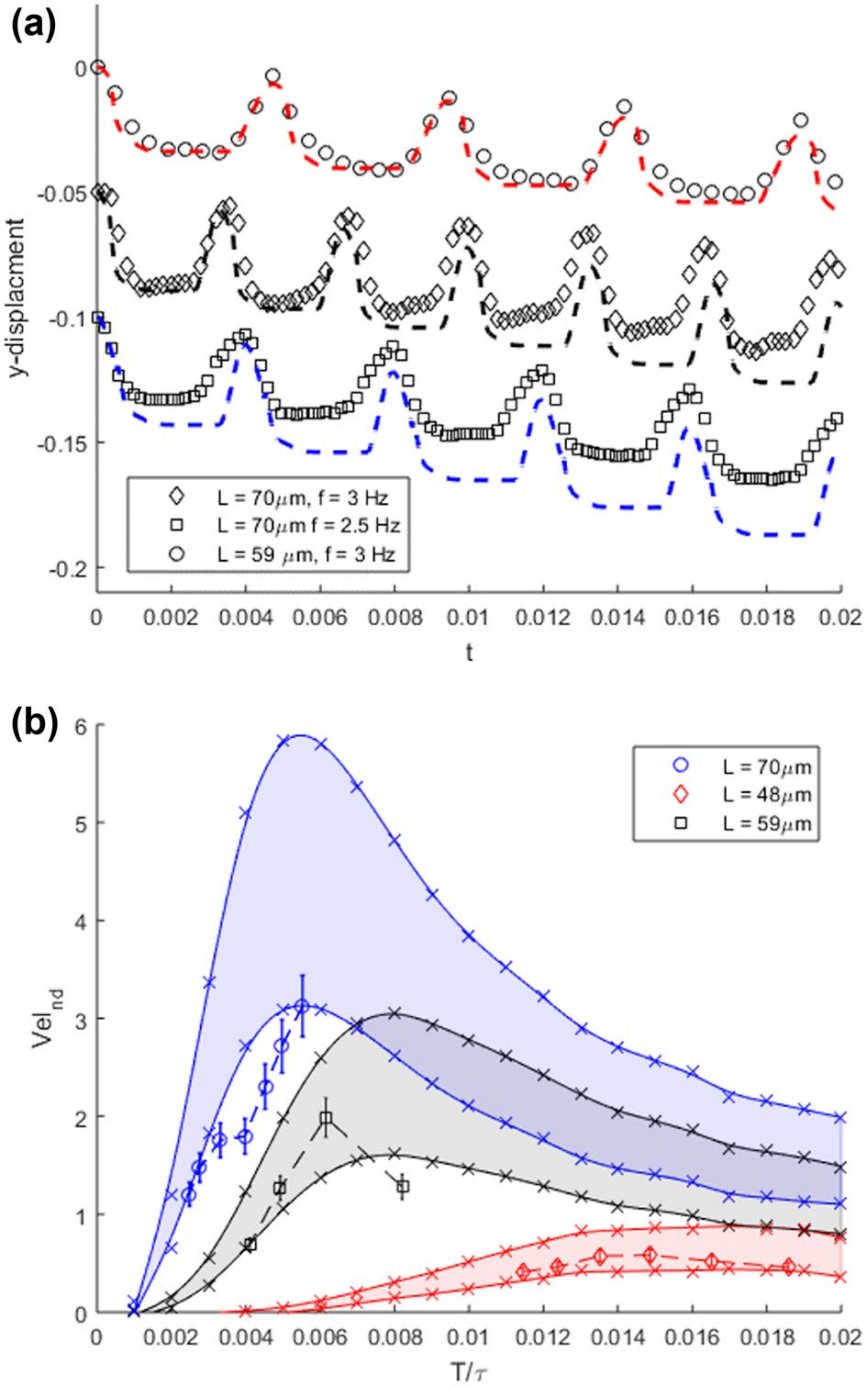
(**a**) Relationship between filament centre of mass y-displacements vs time, circle, diamond and square points donates experimental data. The displacement is scaled by the filament length on the y-axis and with $\tau = 54$ s (diamond and square) and 42 s (circle) on the x-axis. The dashed shows the numerical filament centre with points that correspond to the experiment (black: $Cm = 70$, $T/\tau = 0.0039$), (blue: $Cm = 70$, $T/\tau = 0.031$) and (red: $Cm = 50$, $T/\tau = 0.0047$). (**b**) Scatter points: velocity vs frequency for filaments with lengths $L = 48$ $\upmu$m (diamond), 59 $\upmu$m (circle) and 70 $\upmu$m (square) scaled with L/$\tau$ on y-axis and $\tau$ on the x-axis, operating at a range of frequencies and fixed field strength $H = 5.2$ Oe. The solid lines represent numerical simulations over range of T/$\tau$: $Cm = 70$ (Blue), $Cm = 50$ (Black) and $Cm = 30$ (red). Colored regions boundaries corresponds simulations by defining $\lambda = -0.2$ and $-0.4$ for lower and upper lines respectively.

The swimming behaviour was characterised by the filaments mass center velocity under different operating conditions. The swimming direction was found to be perpendicular to the applied field in the y-direction. Experimentally slight displacements were observed in the x-direction, probably due to the difference in the arms length as mentioned in previously. The y-displacements obtained from filament centre of mass were registered for each time step. The behaviour of the three different experiments can be seen as the diamonds, squares and circles in Fig. 3a scaled by $\tau$ and the filament length. Similarly, the corresponding y-displacements of filament’s median were obtained from numerical simulations as shown in the dashed lines in Fig. 3a. The swimming velocity was then obtained by a linear fit of the y-displacement vs time.

**Figure 4:**
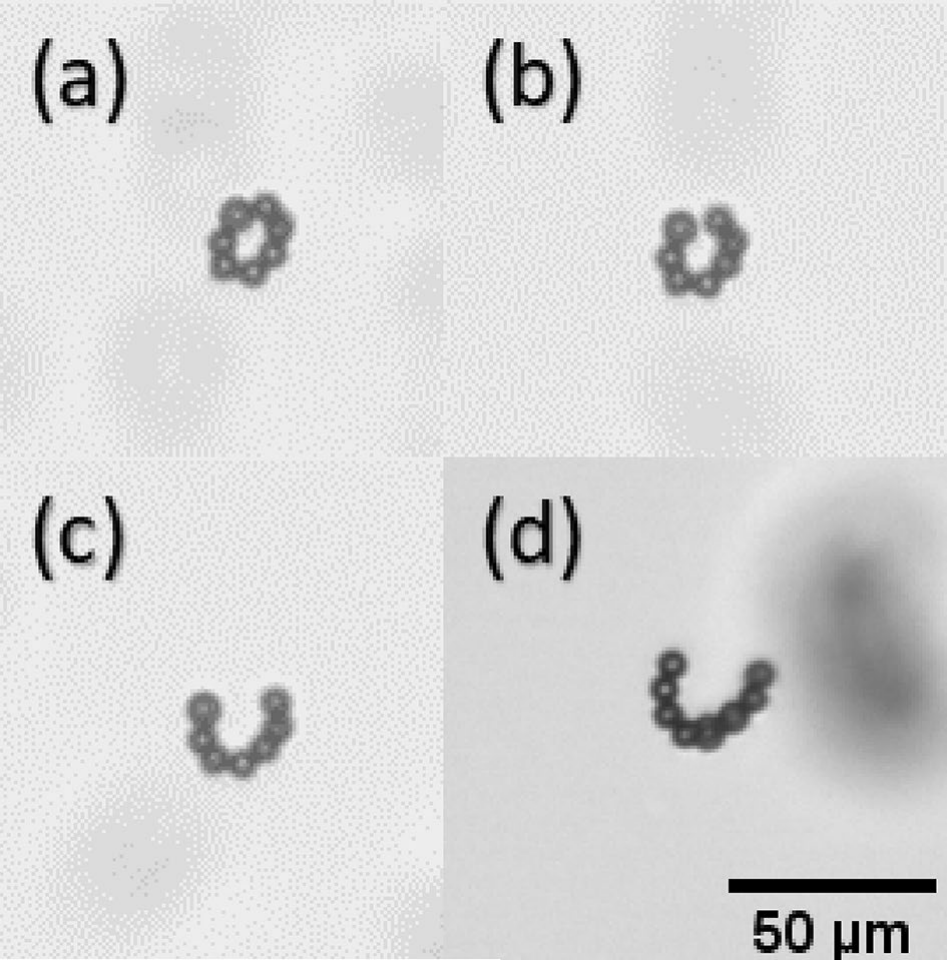
Maximum observed filament deformation within one period for different filaments. In (**a**–**c**) $L = 48$ $\upmu$m, $H = 5.2$ Oe and $f = 4-6$ Hz. In (**d**) $L = 53$ $\upmu$m at fixed $f = 4$ Hz and $H = 5.2$ Oe.

The experiments were repeated for filaments with three different lengths, a range of frequencies and at fixed field strength. The results for the experimental swimming velocity vs frequency are shown in Fig. 3b (data points) and by numerical simulation (solid lines). Here we repeated the simulations using $\lambda$ values of − 0.2 and − 0.4 (lower and upper solid line respectively). It was noticed that for filaments with lengths 48 $\upmu$m and 59 $\upmu$m the velocity reaches to a maximum and then drops. The velocity of 70 $\upmu$m filament was found to decrease in the range of frequencies examined. Due to experimental limitation, the behaviour at lower frequencies could not be registered as the filament ends will get connected to each other and form a ring like shape. An example of a similar ring like shape can be seen in Fig. 4a. Figure 4 shows the maximum observed filament deformation under different operating conditions within one period. The effect of increasing the frequency is shown in Fig. 4a–c for the same filament operating at a fixed field strength; It was observed that the filament deformation decreases as the frequency is increased. The maximum curvature in the middle section decreases when the length is increased, as shown in Fig. 4a,d, at fixed frequency and field strength.

**Figure 5:**
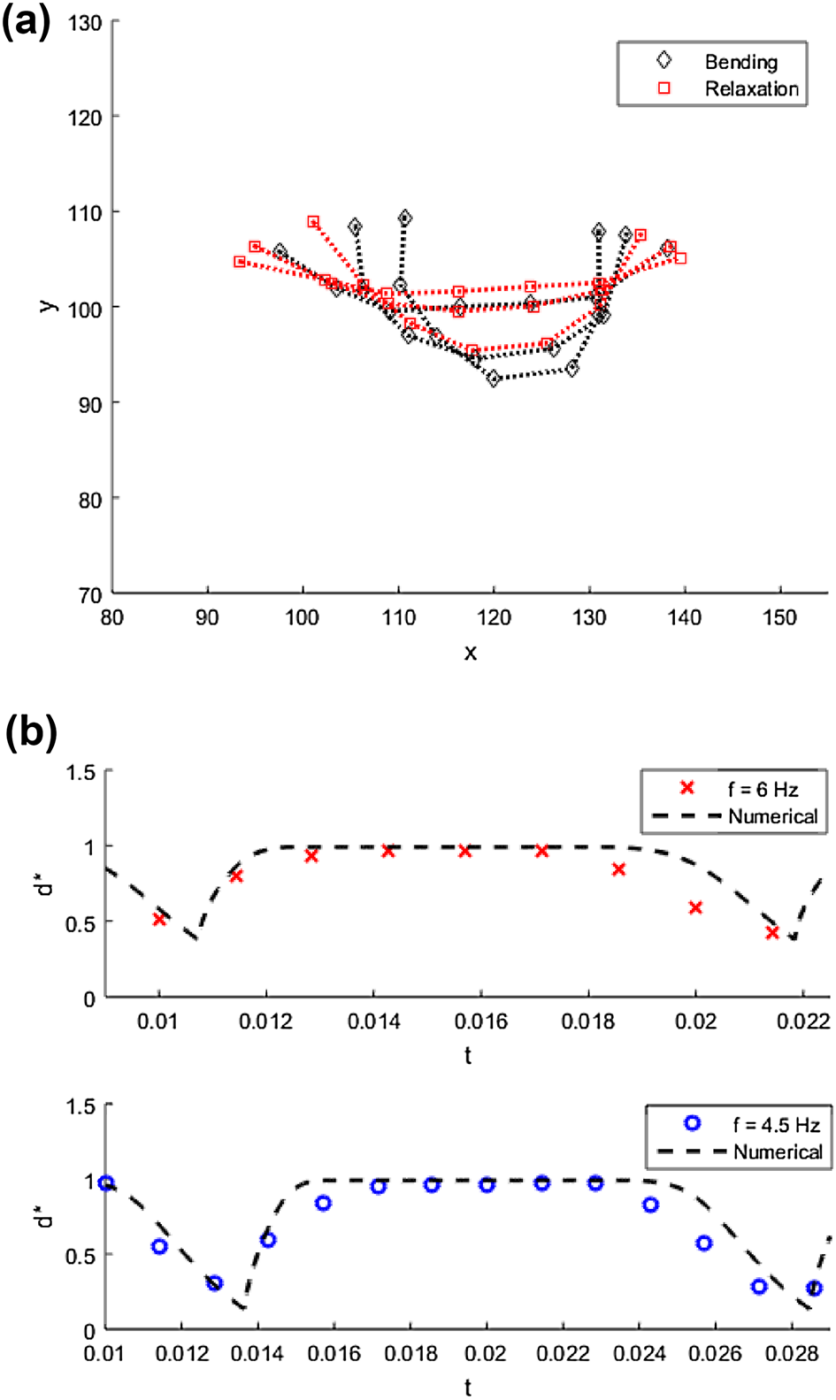
(**a**) Experimental filament configurations showing the difference in bending and relaxation stages at a constant time step over one period. (**b**) End to end x-displacement of filament tips ($d^{*}$) vs time. Scatter points for experimental data for filament with length $L = 48$ $\upmu$m, $H = 5.2$ Oe, frequency $f = 6$ Hz (cross) and $f = 4.5$ (circle). Dashed line shows numerical simulation results of $d^{*}$; $Cm = 30$ , $\lambda = -0.2$, $T/\tau = 0.011$ (upper plot) and $T/\tau = 0.014$ (lower plot).

From the velocity and length ranges obtained shown in Fig. 3b, the calculated Reynolds number is found to be in the magnitude order of $\approx$ $10^{-4}$. Therefore, the propulsion can be assumed to be mainly driven by viscous forces in this regime. By comparing, the experimental filament shapes during bending and relaxation stages at a constant time step, a slight difference of shapes is observed in these stages; resulting in breaking the time reversal symmetry. Thereby satisfying the scallop theorem^26^ to provide propulsion in Stokes hydrodynamics, while the filament velocity is not linearly dependent on the field frequency and is also a function of the difference in filament shapes during bending and relaxation stages. An example of shape difference during bending and relaxation at a constant time step over one period is shown in Fig. 5a. The difference in time between bending and relaxation stages can be seen by registering the difference of x-displacements for both of the filament’s ends $d^{*}$ over time, as shown in Fig. 5b. The experimental results shown in the cross and circle points are scaled by the filament length, while the dashed line shows the corresponding numerical simulations.

We see good agreement between the numerical and experimental results as shown in the figures presented above. Nevertheless, some discrepancies were noticed, which may be due to the small variation of the filament’s physical properties such as the difference in the number of linked DNA strands between the particles and the variation in particle sizes. Moreover, the anisotropy of the hydrodynamic drag coefficient $\lambda$ is length dependent, which can affect both the shape and the velocity in the numerical simulations. The swimming velocity can be optimised by either changing the field profile, as shown in Fig. 5b, in this way the time between the bending and relaxation, where the filament is stationary can be reduced. The field unstable time ratio within one period should be maintained to avoid cessation of swimming and transition to S-shape mode. This transition was numerically investigated in Ref.^18^, and was also observed experimentally. By having the same field profile, the maximum swimming velocity as shown in Fig. 3b, can be achieved by having values of $d^{*}$ close to zero. Moreover, lower values of *Cm* will give a wider range of operating frequency to achieve the maximum velocity, due to reduced flexibility to form a ring-like shape.

## Discussion

Buckling of the ferromagnetic filament in the pulsed magnetic field induces its propulsion. Anisotropy of hydrodynamic drag is essential for this. Quantitative description of experimental data shows that the ratio of the perpendicular and parallel hydrodynamicdrag coefficients is less than 2—a value valid for an infinite straight rod as observed also for different microorganisms. The mechanism of propulsion, as shown by experimental and numerical results, consists of breaking the time inversion symmetry between the bending and straightening stages of the filament. Achieved propulsion velocities by this mechanism are not very high and in the best cases gives a displacement of several percents of the filament length per period, which is much less than, for example, for example, for spermatozoon^27^, which propel at much higher frequencies of deformation. The corresponding frequency range is not accessible for the present filaments since it requires higher field values at which these filaments would break.

To compare the swimming performance of ferromagnetic filaments with those observed for microorganisms^28^, we see that average displacement per period of Chlamydomonas $\Delta x_\text {CR}\approx 2.5$ $\upmu$m is only 3 times larger than for our fastest swimmer ($\Delta x\approx 0.75$ $\upmu$m at $v=3$ $\upmu$m/s and $f=4$ Hz). Thus we believe that the ferromagnetic filament is a demonstration of an artificial puller type microswimmer^29^.

## Methods

### Experimental

The filaments are made from ferromagnetic particles functionalized with strepdavidin linked together by biotinized DNA fragments. The 4.26 $\upmu$m average diameter magnetic particles (Spherotech, $1\%$ w/v) are first mixed with 1000 base pairs long DNA strands (ASLA biotech, biotinated on the 5$^\prime$ ends) in a TE buffer solution ($\eta = 0.01$ P, pH = 7.5). The sample is then placed between two Neodymium magnets (field strength $\approx 500$ Oe) for two minutes to allow the particles to align and form the filaments. More details about the filament formation methodology can be found in Ref.^30^. For sample observation, fluidic cells are used; made from two glass slides and separated by 211 $\upmu$m thick double side adhesive tape. The behaviour of the filaments are registered by optical microscopy (Leica DMI3000B, objectve 10$\times$, bright field mode), via Basler ac1920-155um camera using a fixed acquisition rate of up to 100 Hz. A coil system, which consists of six coils is used to generate the magnetic field. Each coil pair is connected to an AC power supply (Kepco BOP 20-10M). The power supplies are controlled by LabVIEW program via NI-PCI-6229 data acquisition card.

The images are processed using Matlab, the filaments are first segmented from the image by intensity threshold resulting in a binary image. And regionprops function was used to calculate the centroid of the filament. The centreline of the filament is obtained from the particle centre points using circular Hough transform function. The above results for velocity calculation presented are for 500 images processed for each data point, with an acquisition frame rate of 50 fps.

## Supplementary Information

Supplementary Information.

Supplementary Video S1.

Supplementary Video S2.

## Data Availability

Experimental and numerical data used in this study will be published on the open-access repository (https://zenodo.org).

## Code availability

Codes used in this work are available from the authors upon request.
