# Supplementary material for: Instability caused swimming of ferromagnetic filaments in pulsed field
Source: Sci Rep. 2021 Dec 3;11:23399. doi: 10.1038/s41598-021-02541-3 (PMC8642389; doi:10.1038/s41598-021-02541-3)
Supplement: Supplementary file 1 — Supplementary Information. [file 41598_2021_2541_MOESM1_ESM.pdf]

# Instability caused swimming of ferromagnetic filaments in pulsed field

# Supplementary Information

Abdelqader Zaben,[a] Guntars Kitenbergs[a] and Andrejs Cēbers*[a]
[a] *MMML lab, University of Latvia, Jelgavas 3, Riga, LV-1004, Latvia*
* *E-mail: andrejs.cebers@lu.lv*

**Video S1**: An example video of an experimental and numerical swimming filament. (a) Filament with length $L = 48$ $\mu m$, field frequency $f = 5$ Hz and field strength $H = 5.2$ Oe. (b) Filament shapes obtained by numerical simulations that correspond to the filament shown in (a), $Cm = 30$ and $T/\tau = 0.0133$, $\lambda = -0.4$.

**Video S2**: An example of experimentally observed filaments undergoing 's'- like deformation with corresponding numerical simulations. (a) Filament with length $L = 71$ $\mu m$, field frequency $f = 5$ Hz and field strength $H = 5.2$ Oe. (b) and (c): Filament shapes obtained by numerical simulations that correspond to the filament shown in (a), $Cm = 90$ and $T/\tau = 0.0024$, $\lambda = -0.4$.

**Numerical model:**

The numerical model of the ferromagnetic filament is based on the Kirchhoff model of elastic rod including the action of magnetic torques. The magnetic moment per unit length of the rod $\vec{M}$ is antiparallel the local tangent direction $\vec{t}$: $\vec{M} = -M\vec{t}$. The torque balance

$$\frac{d\vec{K}}{dl} + \vec{t} \times \vec{F} - M\vec{t} \times \vec{H} = 0 \tag{1}$$

for the magnetic force gives $\vec{F}_m = M\vec{H}$. The balance of the viscous, elastic and magnetic forces reads

$$\vec{\vec{\zeta}}\frac{\partial \vec{r}}{\partial t} = -A_b \frac{\partial^4 \vec{r}}{\partial l^4} + \frac{\partial(\Lambda \vec{t})}{\partial l} \tag{2}$$

where $\vec{\vec{\zeta}}_{ik} = \zeta_\perp \delta_{ik} + (\zeta_\parallel - \zeta_\perp) t_i t_k$ is the matrix of hydrodynamic drag coefficients, $\Lambda$ is the tension to sustain the inextensibility of the rod and $A_b$ is the bending modulus.

The length is scaled with the length of the filament $L$, time with the elastic relaxation time $\tau = \zeta_\perp L^4/A_b$, the elastic force by $A_b/L^2$. As a result behavior of the rod is determined by the magnetoelastic number $Cm = MHL^2/A_b$ and $\omega\tau$, where $\omega$ is the characteristic frequency of the AC field.

The numerical algorithm is based on the construction of the projection operator projecting the configuration on the class of inextensible configurations. For that the rod is discretized by $\vec{r}_i, i = 1, ...p + 1$ marker points (in simulations $p = 300$ for the most cases). For the inextensible rod there are $p$ constraints $g_i = (\vec{r}_{i+1} - \vec{r}_i)^2 = h^2$. The constraints give $p \times 3(p+1)$ matrix $J_{ij} = \partial g_i / \partial \vec{r}_j$. Approximating the derivatives by finite differences, where the condition that ends are torque free $\partial^2 \vec{r}/\partial l^2 = 0$ is taken into account, the equation of motion introducing $3(p + 1) \times 1$ column vectors $\vec{r}$ and $\vec{F}$ may be written as ($J'$ is transposed matrix)

$$\frac{\partial \vec{r}}{\partial t} = \vec{\vec{\zeta}}^{-1} \cdot (\vec{F} + J' \frac{\Lambda}{2h}) \tag{3}$$

where $\zeta_{ik}^{-1} = \zeta_{\perp}^{-1} + (\zeta_{\parallel}^{-1} - \zeta_{\perp}^{-1}) t_i t_k$ is the mobility matrix. As a result of discretization we obtain the stiffness matrix $A$ and $\vec{F}_k = \vec{F}_k^e = A_{k;l}\vec{r}_l$ for $k = 2, ...p$ and $\vec{F}_{1,p+1} = A_{1,p+1;l}\vec{r}_l + Cm\vec{h}$ for $k = 1$ and $k = p + 1$.

The condition of inextensibility $J \cdot \frac{\partial \vec{r}}{\partial t} = 0$ allows to express $\Lambda$ as follows

$$\frac{\Lambda}{2h} = -(J\zeta^{-1}J')^{-1}J\zeta^{-1}\vec{F} \tag{4}$$

and the equation of motion reads

$$\frac{\partial \vec{r}}{\partial t} = (I - \zeta^{-1}J'(J\zeta^{-1}J')^{-1}J)\zeta^{-1}\vec{F} \tag{5}$$

Eq.(5) contains the projection operator $P = I - \zeta^{-1}J'(J\zeta^{-1}J')^{-1}J$. It is easy to check that $P^2 = P$. Eq.(5) is numerically solved by implicit scheme

$$\frac{\vec{r}(t + \Delta t) - \vec{r}(t)}{\Delta t} = P\zeta^{-1}(\vec{F}^e(t + \Delta t) + \vec{F}_m) \tag{6}$$

After each time-step redistribution of marker points is carried out to sustain their homogeneous distribution along the arclength of the filament's centerline.
